# Middle fossa approach for a facial nerve schwannoma: how I do it

**DOI:** 10.1007/s00701-022-05199-6

**Published:** 2022-04-06

**Authors:** Pablo González-López, Carlos Martorell-Llobregat, Vladimír Beneš, Roy T. Daniel

**Affiliations:** 1 Department of Neurosurgery, Alicante General University Hospital, Alicante Institute of Health and Biomedical Research (ISABIAL), Avda. Pintor Baeza sn, 03010 Alicante, Spain; 2 Department of Neurosurgery and Neurooncology, Military University Hospital and Charles University, First Medical Faculty, Prague, Czech Republic; 3 Department of Neurosurgery, University Hospital of Lausanne and UNIL, Lausanne, Switzerland

**Keywords:** Middle fossa, Tumor, Schwannoma

## Abstract

**Background:**

Facial nerve schwannomas can extend to the middle fossa or the cerebellopontine angle through the labyrinthine and cisternal segments of the facial nerve. The middle fossa approach (MFA) and its extensions provide a wide approach to deal with a large variety of lesions located in the middle and posterior cranial fossa junction.

**Methods:**

We describe the MFA along with its advantages and limitations to treat a facial nerve schwannoma involving the middle and posterior cranial fossa.

**Conclusions:**

The MFA is a well-established route to surgically deal with tumors located in and around the proximal four segments of the facial nerve.

**Supplementary Information:**

The online version contains supplementary material available at 10.1007/s00701-022-05199-6.

## Relevant surgical anatomy

**Fig. 1:**
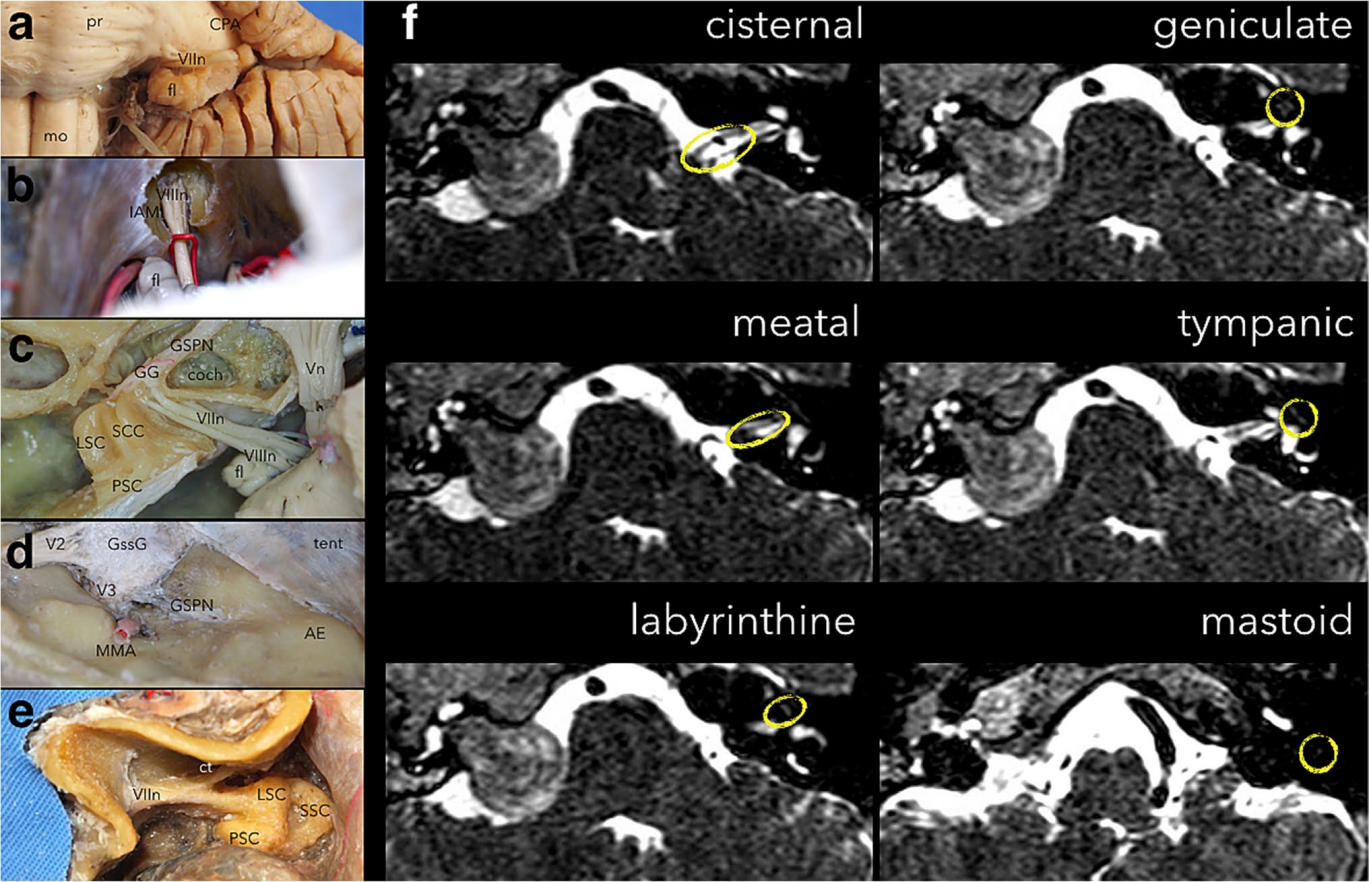
**a** Anterior view of the cerebellopontine angle in the left side of the brainstem showing the cisternal or intracranial segment of the facial nerve. **b** Surgical view in an anatomic specimen showing the cerebellopontine angle view through a retrosigmoid approach after drilling the lateral wall of the IAM and uncovering its contents: vestibular, acoustic, and meatal segment of the facial nerves. **c** Superior view of a left side drilled petrous bone where the close relationship between the meatal, labyrinthine (including geniculate ganglion and GSPN), and tympanic (also described as horizontal segment) segments of the nerve are clearly exposed and related to the labyrinth, cochlea, and the middle ear. **d** Left side middle fossa view in a cadaveric specimen after peeling the dura, highlighting the close relationships between the GSPN, Gasserian ganglion, V3, and the middle meningeal artery. **e** Left side view of the mastoid segment of the facial nerve after a mastoidectomy. Note the relative location of the lateral semicircular canal and exit of the chorda tympani. **f** Axial T2-weigthed images of the cisternal, meatal, labyrinthine, tympanic, and mastoid segment of the facial nerve on the left side, and the large facial nerve schwannoma on the right side. *AE*, arcuate eminence; *coch*, cochlea; *CPA*, cerebellopontine angle; *ct*; chorda tympani; *fl*, flocculus; *GG*, geniculate ganglion; *GSPN*, greater superficial petrosal nerve; *IAM*, internal acoustic meatus; *LSC*, lateral semicircular canal; *MMA*, middle meningeal artery; *mo*, medulla oblongata; *pr*, protuberance; *PSC*, posterior semicircular canal; *SCC*, superior semicircular canal; *tent*, tentorium; *V2*, second division of the trigeminal nerve; *V3*, third division of the trigeminal nerve; *VIIn*, facial nerve; *VIIIn*, vestibulocochlear nerve; *Vn*, trigeminal nerve

The facial nerve segments and their relationship with structures within the temporal bone should be studied to understand the anatomy of the MFA. The facial nerve is divided into six segments: cisternal or intracranial, meatal, labyrinthine, tympanic, vertical or mastoid, and extracranial or intraparotid segments. Facial schwannomas can arise from any of these segments of the nerve (Fig. 1). Nevertheless, it has been noted that there is a predilection for facial nerve schwannomas to arise from the perigeniculate area [3, 6, 10].

**Fig. 2:**
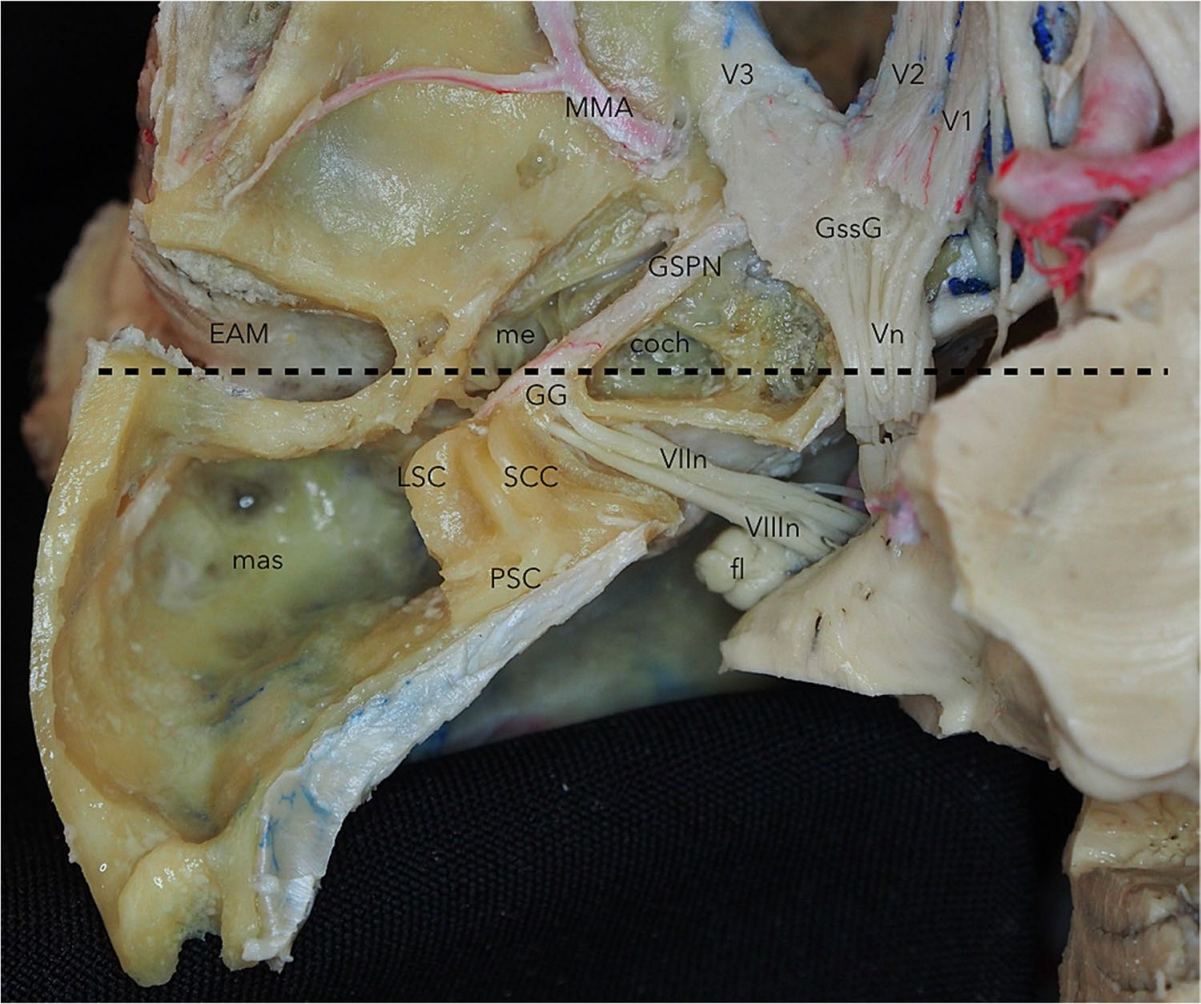
Superior view of the left middle fossa after peeling the dura away. The mastoid has been widely drilled, as well as the roof of the external and internal auditory meatus. The labyrinth has been drilled and the semicircular canals skeletonized. The cochlea is shown anterior to the junction of the meatal segment of the facial nerve and the geniculate ganglion. The acoustic nerve is entering the cochlea anteriorly in the fundus of the IAM, while the superior vestibular nerve is shown moving posteriorly to the labyrinth. The short tympanic segment of the facial nerve is exposed on its middle ear path. The black-dotted line shows a vertical plane dividing the middle fossa floor into two regions, passing over the anterior border of the cochlea. The posterior part includes the bony labyrinth, which consists of the vestibule, cochlea, and superior semicircular canal (it projects toward the middle fossa floor forming the arcuate eminence). *coch*, cochlea; *EAM*, external acoustic meatus; *fl*, flocculus; *GG*, geniculate ganglion; *GSPN*, greater superficial petrosal nerve; *GssG*, Gasserian ganglion; *LSC*, lateral semicircular canal; *mas*, mastoid; *MMA*, middle meningeal artery; *me*, middle ear; *PSC*, posterior semicircular canal; *SCC*, superior semicircular canal; *V1*, first division of the trigeminal nerve; *V2*, second division of the trigeminal nerve; *V3*, third division of the trigeminal nerve; *VIIn*, facial nerve; *VIIIn*, vestibulocochlear nerve; *Vn*, trigeminal nerve

Rhoton [10] separated the middle fossa floor into two regions, divided by a vertical plane passing over the anterior border of the cochlea. The posterior part includes the bony labyrinth, which consists of the vestibule, cochlea, and superior semicircular canal (it projects toward the middle fossa floor forming the arcuate eminence). These structures are closely related to the meatal, labyrinthine, and tympanic segments of the facial nerve and the geniculate ganglion. The posterior component of the fossa is intimately related to the lateral part of the internal auditory canal. Anteriorly, the petrous internal carotid artery (ICA), the Eustachian tube, and the tensor tympani are found deep and parallel to the GSPN. The bony thickness covering these structures is highly variable. Therefore, an accurate preoperative analysis of the images is mandatory to determine the bony cover for the petrous segment of the ICA. The GSPN emerges from the geniculate ganglion and passes through the sphenopetrosal groove along the middle fossa floor, traveling anterior to the level of V3 segment of the trigeminal nerve, where it passes under the nerve [1, 3, 8, 10] (Fig. 2). The intersection of the GSPN with the trigeminal nerve, the porus trigeminus; the intersection of the arcuate eminence, and petrous ridge; and the intersection of the lines projected along the axes of the GSPN and arcuate eminence were described by Day et al. as the “rhomboid construct” limits to safely drill this area and increase the posterior fossa access [2].

Facial nerve tumors, in both the middle and posterior cranial fossa, are confined within the layers of dura or are “interdural” in its location. This dural layer provides a reliable, safe plane for surgical dissection and protects all the adjoining critical vascular and neural structures.

**Fig. 3:**
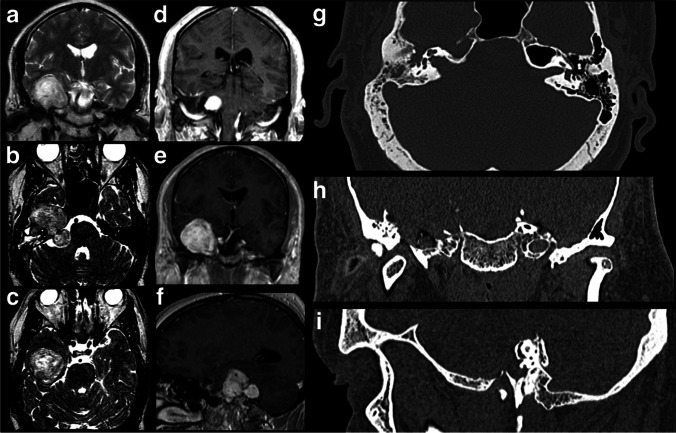
**a**–**c** Preoperative T2-weighted coronal and axial MRI cuts showing a relatively hyperintense heterogeneous well-circumscribed tumor, occupying the floor of the middle fossa, internal acoustic meatus, and posterior fossa CPA. It shows the classic dumbbell shape of a middle-posterior fossa facial nerves schwannoma. **d**–**f** Preoperative T1-weighted after gadolinium injection, showing the homogeneous enhancement of the different areas of the tumor. **g**–**i** Preoperative bone window CT scan axial, coronal, and sagittal cuts showing the huge erosion caused by the tumor in the middle fossa floor structures. The internal auditory meatus is widely enlarged by the tumor, while the petrous segment of the internal carotid artery is deprived of its bone cover

In this report, the MFA is demonstrated in a patient with a facial schwannoma with middle and posterior cranial fossa extension, presenting with headache, ipsilateral trigeminal neuralgia, House-Brackmann grade V facial palsy, and hearing loss (Fig. 3). The surgical access to the internal auditory canal was accurately described more than half a century ago by House [5].

## Description of the technique

### Positioning

**Fig. 4:**
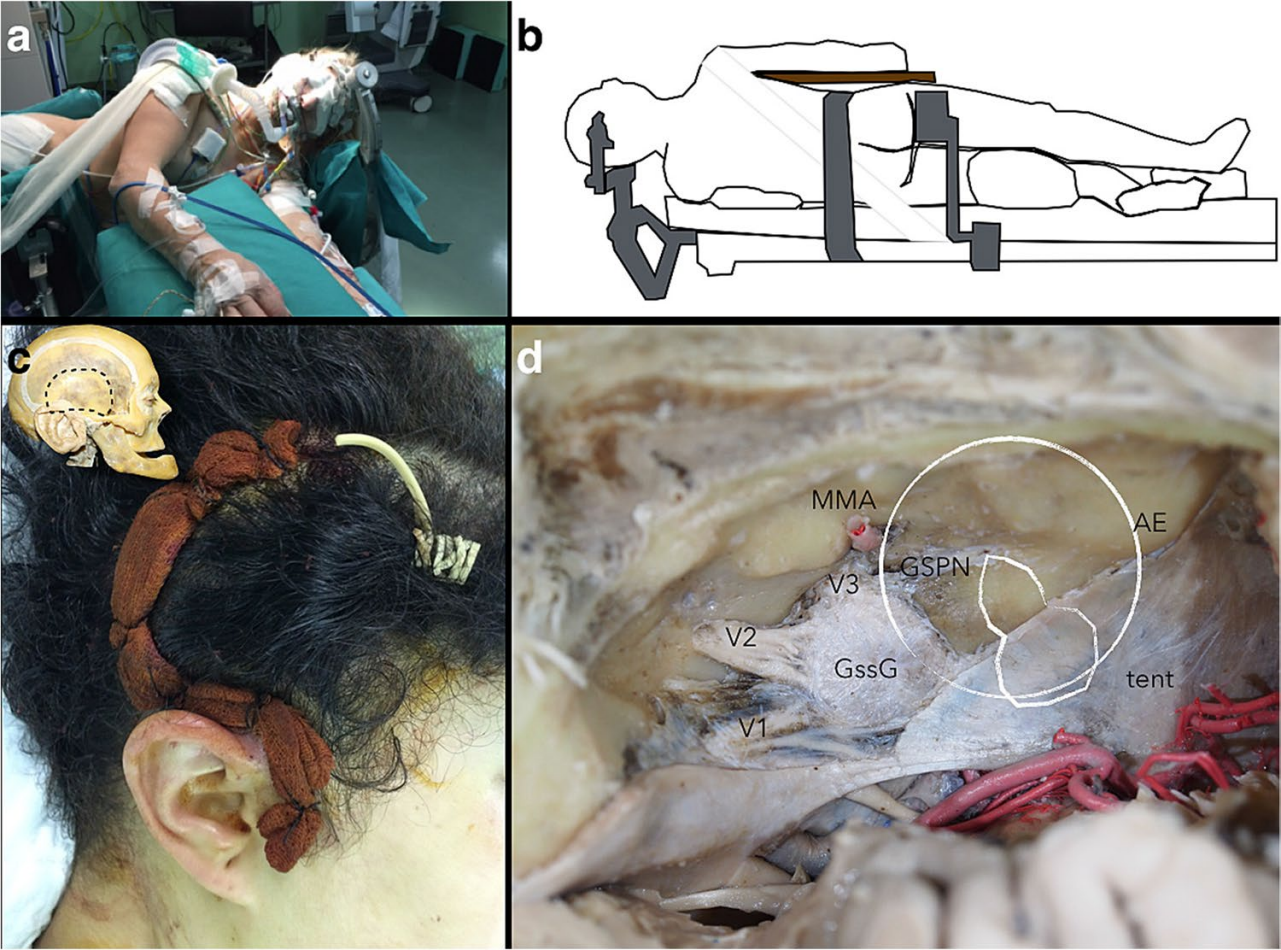
**a**, **b** Left lateral position. The head is slightly extended towards the contralateral side, while the ipsilateral shoulder is retracted downwards to increase the surgical working space. **c** Question mark parieto-temporal skin incision. **d** Simulation of the surgical view on a cadaveric specimen showing the relevant anatomical structures after peeling the middle fossa floor dura. The tumor relative location is drawn (*white*). *AE*, arcuate eminence; *GSPN*, greater superficial petrosal nerve; *GssG*, Gasserian ganglion; *MMA*, middle meningeal artery; *tent*, tentorium; *V1*, first division of the trigeminal nerve; *V2*, second division of the trigeminal nerve; *V3*, third division of the trigeminal nerve

The patient is placed in lateral position. The head is slightly extended towards the contralateral side, while the ipsilateral shoulder is retracted downwards to increase the surgical working space. A lumbar drainage is used to extract 15–30 cc during the craniotomy. Intraoperative neuromonitoring is used (Fig. 4).

### Skin incision

A parieto-temporal question mark–shaped incision is commonly employed. Fascia and temporalis muscle are exposed and retracted anteriorly with a soft tissue curved self-retractor exposing the posterior root of the zygoma, above which, a burr-hole is made to ensure exposure of the middle fossa.

### Craniotomy and epidural access

A craniotomy, one-third behind and two-thirds anterior to the external auditory meatus, is performed (Fig. 4). A high-speed drill is used for the removal of the temporal bone border until the inferior margin of the craniotomy reaches the middle fossa floor. An extradural subtemporal approach is utilized to expose the tumor, which was confined within the layers of dura (periosteal and propria). This dural cover provides a safe plane for surgical dissection (“interdural approach”) [5].

### Resection

A transverse incision is made on the dura that covers the tumor and then it is debulked using the ultrasonic aspirator. Special care must be given to the petrous segment of the ICA. The small probe vascular doppler is used to localize it and to avoid blind dissection maneuvers at that point. Once a macroscopic complete resection of the middle cranial fossa component is achieved, a diamond drill with continuous irrigation is used to expose the IAM. Drilling starts medially at the petrous ridge in a line bisecting the angle between the superior semicircular canal (arcuate eminence) posteriorly and the GSPN anteriorly. Drilling continues posterolaterally toward the geniculate ganglion. The angle between the long axis of the GSPN and the internal acoustic meatus (IAM) can be used to locate the ICA. The posterior fossa dura is opened medial to lateral after coagulating and sectioning the superior petrosal sinus. This maneuver facilitates the exposure of the tumor in relation to the cisternal, meatal, and labyrinthine segments of the nerve. Partial resection of the cisternal component of the tumor was achieved. Special care is driven to achieve an adequate decompression of the trigeminal nerve after tumor removal.

### Closure

**Fig. 5:**
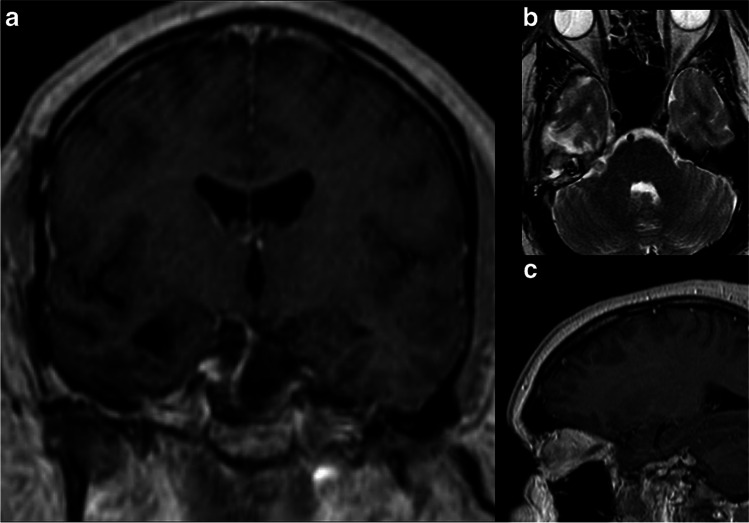
**a** Postoperative T1-weighted coronal MRI cut after gadolinium injection, showing a complete resection of the middle fossa component. **b** Postoperative T2-weighted MRI cut showing mild edema in the basal aspect of the temporal lobe, as well as a small tumor remnant in the cerebellopontine angle. **c** Postoperative T1-weighted sagittal MRI cut after gadolinium injection, showing a complete resection of the middle fossa and intrameatal components, and a small tumor remnant in the posterior fossa

After achieving hemostasis, the floor of the middle fossa is reconstructed with a fibrin sealant patch. Bone wax is used to seal the drilled areas of the middle fossa to avoid postoperative CSF leak. In case a large defect has been created after drilling the middle fossa bone, autologous fat is a great option to prevent CSF leaks. The bone flap is repositioned and fixed with screws, while the muscle and skin are closed in the regular way. The postoperative period remained uneventful with a complete relief of the trigeminal pain; there were no changes in the preoperative facial nerve paralysis (Fig. 5).

## Indications

The MFA offers an excellent approach to deal with a variety of pathological lesions located at the junction of the middle and posterior cranial fossa [7]. The MFA and its variations are a well-established route to reach the proximal 4 segments of the facial nerve, the GSPN, and the geniculate ganglion [10]. It can be used in patients with large intracanalicular tumors, lesions located in the IAM with cisternal extension of < 1.5 cm, and tumors extending to the fundus of the IAM [1, 9]. This technique has undergone several modifications to expand its exposure along the cerebellopontine angle, petrous apex, tentorial incisura, upper clivus, and posterior cavernous sinus [7, 10].

## Limitations

The main limitations of the MFA include the risk of seizures and possible language impairment in the dominant hemisphere secondary to excessive retraction of the temporal lobe, the difficulty in resecting larger tumors, the narrow surgical corridor offered, and the tight exposure of posterior fossa, which might limit the resection of lesions with a significant cisternal component projecting downwards to the lower CPA floor. The vestibule and the cochlea can be injured during drilling of the bone above the labyrinthine segment of the facial nerve at the lateral part of the IAM [1, 4, 9, 10].

## How to avoid complications

To prevent postoperative CSF leak, the air cells in the lower lateral part of the middle fossa floor and inner ear should be adequately sealed. If the middle ear is opened, the tegmen must be reconstructed properly to avoid a conductive hearing loss or a CSF fistula. Bipolar cautery of any blood vessel in the IAM should be avoided to minimize the risk of thermal damage to the labyrinthine artery and cochlear and facial nerves. Gentle retraction of the temporal lobe should be done to avoid the intraoperative seizures and a postoperative language impairment. Intraoperative neuromonitoring must be used to identify and secure the cranial nerves. Special care must be taken during drilling of the bone above the labyrinthine segment of the facial nerve to avoid the injury to the vestibule and the cochlea [1, 3, 10].

## Specific information for the patient

The patient should know that facial paralysis will probably not improve after surgery as well as the possibility of a new hearing impairment [4]. Moreover, the need for cranial nerve manipulation and the use of intraoperative monitoring should be explained, as well as the possibility of partial resection to avoid injury to adjacent neurovascular structures if safe dissection is not possible.

## Supplementary Information

Below is the link to the electronic supplementary material.

Supplementary file1 (MOV 90508 KB)
